# Contribution of Phenolics and Free Amino Acids on the Antioxidant Profile of Commercial Lemon Verbena Infusions

**DOI:** 10.3390/antiox12020251

**Published:** 2023-01-22

**Authors:** Juliana A. Barreto Peixoto, Gerardo Álvarez-Rivera, Anabela S. G. Costa, Susana Machado, Alejandro Cifuentes, Elena Ibáñez, M. Beatriz P. P. Oliveira, Rita C. Alves

**Affiliations:** 1 Requimte/LAQV, Department of Chemical Sciences, Faculty of Pharmacy, University of Porto, R. J. Viterbo Ferreira, 4050-313 Porto, Portugal; 2 Laboratory of Foodomics, Institute of Food Science Research, CIAL, CSIC, Nicolas Cabrera 9, 28049 Madrid, Spain

**Keywords:** *Aloysia citriodora*, *Lippia citriodora*, *Aloysia triphylla*, antioxidant activity, UHPLC-QTOF-HRMS, RP-HPLC-FLD, geographical origin, packaging

## Abstract

Lemon verbena infusions are widely appreciated due to their agreeable lemony flavor and medicinal properties. In this study, the antioxidant potential, phenolic profile, and free amino acid profile of lemon verbena infusions from different commercial brands were studied. Characterization by UHPLC-QTOF-HRMS allowed the identification of 34 phenolics. The free amino acid profile (by RP-HPLC-FLD) was assessed for the first time, allowing the quantification of 16 amino acids. Furthermore, the infusions showed high antioxidant activity by different assays (ferric reducing antioxidant power, DPPH^•^ scavenging, and oxygen radical absorbance capacity assays), which in turn were significantly correlated with total phenolics and total flavonoid contents. Notwithstanding, phenylalanine seemed to have also an impact on the antioxidant activity of the infusions, with significant correlations found. Finally, significant differences were found in all the evaluated parameters for one of the four commercial brands herein studied, which was possibly related to the different geographical origins of this sample. Overall, these lemon verbena infusions proved to be rich in a huge variety of bioactive compounds that can provide therapeutic potential.

## 1. Introduction

Lemon verbena, scientifically known as *Aloysia citriodora* Paláu, *A. citridora* Paláu, *Lippia citriodora* Kunth, *L. citrodora* Kunth, *A. triphylla* (L’Hér.) Britton, or *L. triphylla* (L’Hér.) Kuntze [1] is a shrub belonging to the Verbenaceae family widely recognized as a medicinal plant due to a variety of pharmacological activities well-documented in the literature: digestive, antispasmodic, diuretic, neuroprotective, anticonvulsant, anxiolytic, anti-nociceptive, anti-inflammatory, antipyretic, antimicrobial, anticancer, cardioprotective, and antioxidant, among others [1,2]. In fact, lemon verbena is traditionally consumed in the form of an infusion for the treatment or relief of some gastrointestinal disorders (e.g., flatulence, colic, indigestion), insomnia, fever, cold, and asthma [1,2,3]. Many of these properties are largely attributed to phenolic compounds [4]. In particular, verbascoside is considered the main bioactive compound of this plant, presenting important biological activities related to its antioxidant activity, including anti-inflammatory, antitumoral, antimicrobial, and neuroprotective [1,5]. Notwithstanding, several studies have already demonstrated that the biological activities of lemon verbena extracts—namely, the antioxidant capacity—are better than those found for isolated verbascoside [6,7,8]. Thus, synergistic interactions between different compounds present in the whole plant extract seem to provide an enhanced bioactive potential compared with the isolated compound of the plant.

Besides phenolics, other compounds in lemon verbena have been identified and documented as responsible for some of their bioactive properties. For example, several monoterpenes, sesquiterpenes, and iridoid glycosides were demonstrated to possess antimicrobial, antiviral, immunomodulator, anesthetic, neuroprotective, insecticidal, and anticancer activities [1,9]. However, many other unknown compounds might also be present in lemon verbena, and together with the described phytochemicals, they might enhance the biological potential of this plant. As far as we know, no studies on the amino acid profile of this plant have yet been conducted. In addition to being essential for the synthesis of proteins, peptides, and secondary metabolites (such as phenolic compounds) that play important roles in plant adaptation and protection from environmental conditions, these primary metabolites are also endowed with important bioactive properties, particularly antioxidant ones [10,11,12]. Indeed, several studies have demonstrated that some amino acids (e.g., tryptophan, tyrosine, phenylalanine, aspartate, glutamate, histidine, proline, asparagine, cysteine, alanine, isoleucine, and methionine) can act as efficient antioxidants by various mechanisms of action, namely by scavenging free radicals, reducing hydroperoxides, chelating pro-oxidative transition metals, or by acting as biomarkers of oxidative stress [10,11,12]. In this way, the study of the composition of plants in free amino acids may be an important tool to better characterize their biological potential and to open a range of opportunities for their valorization and the development of new plant-based products enriched with this type of bioactive compounds.

In the present study, we aimed to assess the antioxidant properties and the total contents in phenolic and flavonoid compounds of lemon verbena infusions prepared with four different commercial brands, as well as to achieve a comprehensive chemical characterization of these infusions regarding phenolic compounds and free amino acids. The phenolic profile was analyzed by ultra-high-performance liquid chromatography coupled to high-resolution quadrupole/time-of-flight mass spectrometry (UHPLC-QTOF-HRMS), and the free amino acid profile by automatic online pre-column derivatization/reversed-phase high-performance liquid chromatography coupled to fluorescence detection (RP-HPLC-FLD). As far as we know, this is the first study that assesses the free amino acid profile of lemon verbena infusions. Furthermore, the different samples were compared to each other in qualitative and quantitative terms in order to ascertain if the uniformity of the herbal products commercially available is (or is not) guaranteed to consumers.

## 2. Materials and Methods

### 2.1. Reagents and Standards

For in vitro antioxidant activity assays, 1,1-diphenyl-2-picrylhydrazyl radical (DPPH^•^), 6-hydroxy-2,5,7,8-tetramethylchroman-2-carboxylic acid (Trolox), 2,4,6-tripyridyl-s-triazine (TPTZ), sodium acetate, ferric chloride, ferrous sulfate, sodium fluorescein, 2,2′-azo-bis,2-amidinopropane dihydrochloride (AAPH), sodium carbonate, sodium nitrite, aluminum chloride, gallic acid, and catechin were acquired from Sigma-Aldrich (St. Louis, MO, USA), whereas glacial acetic acid, hydrochloric acid 37%, Folin–Ciocalteu’s reagent, and sodium hydroxide were from Merck (Darmstadt, Germany). For phenolics characterization by UHPLC-ESI-QTOF-MS, acetonitrile was purchased from VWR Chemicals (Barcelona, Spain), formic acid was from Honeywell Riedel-de Haën (Seelze, Germany), and methanol and chloroform were from Carlo Erba Reagents (Barcelona, Spain). Finally, for free amino acid analysis by RP-HPLC, individual amino acids, L-norvaline, and the Amino Acids Mix Solution (certified reference material, Trace CERT^®^), were all purchased from Sigma-Aldrich (St. Louis, MO, USA). HPLC-grade acetonitrile and methanol, and sodium azide (99%) were achieved from Honeywell Riedel-de Haën TM (Seelze, Germany), while borate buffer (0.4 N, pH 10.2) and the derivatization reagents, *o*-phtalaldedehyde/3-mercaptopropionic acid (OPA/3-MPA) and 9-fluorenylmethyl chloroformate (FMOC), were from Agilent Technologies (Santa Clara, CA, USA). Ultrapure water was prepared in a Millipore System (Bedford, MA, USA). All other reagents were of analytical grade.

### 2.2. Samples

Four different commercial brands selling lemon verbena were selected and acquired, two of them from two distinct supermarkets (brands A and C) and the other two from two distinct herbalists (brands B and D). The selected commercial brands indicated in their label the scientific name of the species (mentioned as *Lippia citriodora* in all brands) as well as their common name in Portugal (“lúcia-lima” and/or “limonete”). Further, the country of origin of all samples was also present on their label. Lemon verbena of commercial brand A was from Spain, while the remaining ones were from Portugal. Aside from that, commercial brand A presented their products available in bulk, while the others were available in packages. In order to try to guarantee the consistency of the results as much as possible, the selected samples had the same form of presentation (aerial parts of dried plant with similar-sized fragments) and were intended to be consumed as an infusion. Despite brand A being sold in bulk, it was not excluded from the study because we also considered it important to investigate the possible influence of external factors on the parameters investigated in this study. The acquired samples had the same expiry date. After acquisition, they were stored at room temperature and protected from light until infusions preparation.

### 2.3. Infusions Preparation

The infusions were prepared as previously described [13]. Briefly, 200 mL of boiling water was added to 1 g of sample, followed by a rest at room temperature for 5 min, with two agitations during this time. The infusions were then filtered, and aliquots were taken and stored at −21 °C until analyses. For each commercial brand, infusions were prepared in triplicate.

### 2.4. In Vitro Antioxidant Activity

#### 2.4.1. DPPH^•^ Radical-Scavenging Activity

The DPPH^•^ scavenging activity of the infusions herein studied was performed according to Peixoto et al. [13]. Very briefly, 30 µL of the sample was mixed with an ethanolic solution containing DPPH^•^ radicals (6 × 10^−5^ M). After 20 min, absorption was measured at 525 nm. The results were expressed as µg of Trolox equivalents (TE) per mL of infusion. The assay was executed in triplicate.

#### 2.4.2. Ferric Reducing Antioxidant Power (FRAP)

The reducing power of the samples was assessed as previously described by Peixoto et al. [13] with minor modifications. Briefly, 35 µL of the sample (diluted at 1:10 for commercial brand A and 1:100 for commercial brands B, C, and D) were reacted with the FRAP reagent and, after incubation (37 °C, 30 min), the increase in absorbance was measured at 595 nm. The results were expressed as µg of ferrous sulfate equivalents (FSE) per mL of infusion. The assay was performed in triplicate.

#### 2.4.3. Oxygen Radical Absorbance Capacity (ORAC)

The ability of the different studied infusions to act as scavengers against ROO^•^ was evaluated as described by Peixoto et al. [13] with minor modifications. In brief, 25 µL of the sample (diluted at 1:10 for commercial brand A, 1:50 for commercial brand B, and 1:20 for commercial brands C and D) were mixed with fluorescein, followed by incubation (37 °C, 30 min). Then, AAPH was added to the mixture, and fluorescence (λ_exc_ = 480 nm and λ_em_ = 520 nm) was measured every 2 min for 2 h. After this time, the net area under the curve (AUC) of the standard (Trolox) and samples were calculated. A calibration curve was obtained by plotting the net AUC of different concentrations of the standard against the average net AUC of the measurements for each concentration, and the results were expressed as µg of Trolox equivalents (TE) per mL of infusion. The assay was performed in triplicate.

#### 2.4.4. Determination of Total Phenolic Content

The determination of the total content of phenolic compounds in lemon verbena infusions was executed according to Peixoto et al. [13]. In brief, 30 µL of the sample (diluted at 1:10 for commercial brands B, C, and D) were added to the Folin–Ciocalteu reagent (1:10) and sodium carbonate solution (7.5% m/v), followed by incubation (45 °C, 15 min). Absorbance was then recorded at 765 nm. The total phenolics content was expressed as µg of gallic acid equivalents (GAE) per mL of infusion. The assay was performed in triplicate.

#### 2.4.5. Determination of Total Flavonoids Content

The total content of flavonoids in the infusions was determined based on the method reported by Peixoto et al. [13]. Very briefly, 1 mL of sample (diluted at 1:5 for all commercial brands), distilled water, and sodium nitrite (5%) were mixed and incubated for 5 min. Then, aluminum chloride (10%) was added, and 1 min later, sodium hydroxide (1 M) and distilled water were both added. Absorbance was then recorded at 510 nm. The total flavonoid content was expressed as µg of catechin equivalents (CE) per mL of infusion. The assay was performed in triplicate.

### 2.5. Analysis of Phenolic Compounds by UHPLC-ESI-QTOF-MS

The phenolic composition of the infusions was analyzed as previously described [13]. Briefly, after lyophilization, dry residues of lemon verbena infusions were resuspended in methanol/water (50:50, *v*/*v*) and filtered (0.22 µm). The obtained extracts were analyzed in an Agilent 1290 UHPLC system (Agilent Technologies, Santa Clara, CA, USA) coupled to an Agilent 6540 quadrupole-time-of-flight mass spectrometer (Q-TOF MS) equipped with an orthogonal ESI source. The chromatographic separation was performed using a Zorbax Eclipse Plus C18 column (2.1 × 100 mm, 1.8 μm of particle diameter; Agilent Technologies, Santa Clara, CA, USA). The phenolic compounds were then tentatively identified using the Agilent Mass Hunter Qualitative analysis software (version B.07.00, Agilent Technologies, Santa Clara, CA, USA), making use of accurate mass data, ion source fragmentation, MS/MS fragmentation patterns, MS databases (e.g., HMDB, Metlin, PubChem), and bibliographic search. For quantitative comparison in terms of relative abundance, peak area values were obtained using the Agilent Mass Hunter Quantitative analysis software (for Q-TOF, version B.08.00, Agilent Technologies, Santa Clara, CA, USA).

### 2.6. Analysis of Free Amino Acids by RP-HPLC-FLD

The analysis of the free amino acid composition of the different lemon verbena infusions was performed based on a methodology recently developed by Machado et al. [14]. Briefly, 1.5 mL aliquots of the infusions were centrifuged at 13,000 rpm for 10 min in a Heraeus Fresco 17 centrifuge (Thermo Fisher Scientific, Schwerte, Germany). Then, 990 µL of supernatant were collected in a new eppendorf and mixed with 10 µL of the internal standard (norvaline, 2 mg/mL). The mixture was then transferred into a 2 mL injection vial and put into the autosampler. All the procedures were carried out in triplicate. The chromatographic analysis was performed in an integrated system from Jasco (Tokyo, Japan) equipped with an LC-NetII/ADC hardware interface, two Jasco PU-980 intelligent HPLC pumps, a high-performance autosampler (Jasco AS-4150 RHPLC autosampler), and a fluorescence detector (Jasco FP-2020 Plus). The oven column heater was from Jones Chromatography (Model 7981, Hengoed, UK). The samples were derivatized with OPA/3-MPA and FMOC reagents using an automatic online derivatization procedure [14]. Amino acids were separated in a ZORBAX Eclipse Plus C18 (4.6 × 250 mm, 5 µm) column from Agilent Technologies (Santa Clara, CA, USA), kept at a constant temperature of 40 °C using a gradient solvent system at a flow rate of 1.5 mL/min, according to Machado et al. [14]. Fluorescence detection was monitored for OPA-derivatives at λ_exc_ = 340 nm/λ_em_ = 450 nm (from 0.0 to 26.2 min) and for FMOC-derivatives at λ_exc_ = 266 nm/λ_em_ = 305 nm (from 26.2 to 40.0 min). The chromatograms obtained were analyzed with the JASCO-ChromNAV software (version 2.02.08, Jasco, Tokyo, Japan), and the amino acids were identified based on the retention time of the respective standards. The quantification of each amino acid was based on the response of the fluorescence signal of each standard, converted into units of concentration through calibration curves obtained for each amino acid using the standard internal method. The results were expressed in ng of amino acid per mL of infusion.

### 2.7. Statistical Analysis

The SPSS Statistics 26 for Windows program (IBM Corp., Armonk, NY, USA) was used to analyze the results, which were expressed as mean ± standard deviation. A one-way ANOVA test was used to assess differences between samples, followed by post hoc comparisons through Tukey’s HSD test. The results were considered statistically significant when *p* < 0.05. Moreover, the existence of linear relationships between parameters was evaluated by Pearson’s correlation tests.

## 3. Results and Discussion

### 3.1. Antioxidant Activity and Total Contents in Phenolics and Flavonoids

The antioxidant activity and total contents of phenolic and flavonoid compounds of the different commercial lemon verbena infusions are presented in Table 1. The samples showed high antioxidant activity in all assays, as well as high contents of total phenolic and flavonoid compounds. In general, our results are in agreement with those reported in some studies: on the one hand, the total phenolic contents were 2-fold higher than those reported by Jimenez-Zamora et al. [15] and approximately 7-fold higher than those reported by Gião et al. [16]; on the other hand, our antioxidant activity results were similar to those described by Sanchez-Marzo et al. [17] in ORAC assay but 4-fold lower than those experimented by Jimenez-Zamora et al. [15] in the DPPH^•^ scavenging assay. These variations in the results might be explained by the extraction method implemented in each study, the part of the plant used (e.g., Gião et al. [16] used only the leaves, while, in our study and in the study conducted by Jimenez-Zamora et al. [15], the various aerial parts of the plant were used), and to the different procedures applied by different laboratories, mainly in the DPPH^•^ scavenging assay. Notwithstanding, the overall results were quite positive. In addition, comparing our results with those obtained in two previous studies carried out with cherry stem infusions and rosemary infusions in our laboratory [13,18], lemon verbena showed an antioxidant potential significantly more expressive (compared with the average values of cherry stem infusions: DPPH^•^ scavenging assay was 4-fold higher, FRAP assay was 8-fold higher, ORAC assay and total phenolic content were 6-fold higher, and total flavonoid content was 9-fold higher; compared with the average values of rosemary infusions: DPPH^•^ scavenging assay was 3-fold higher, FRAP and ORAC assays were 6-fold higher, and total phenolic and total flavonoid contents were 7-fold higher).

The lemon verbena from brand A was sold in bulk and cultivated in Spain, while the remaining ones were sold in packages and were all cultivated in Portugal, and the results seem to reflect those differences. Indeed, the infusions from brand A presented significantly lower values (*p* < 0.05) in all the spectrophotometric assays in comparison with the infusions prepared with the three remaining brands. These discrepant differences may have different explanations. On the one hand, the lack of packaging of samples from brand A might have influenced the low results obtained, thus suggesting that this plant, and particularly the phenolic compounds present in it, might have been subjected to oxidations resulting from the exposure to external factors—such as light, heat, and moisture—as was also previously observed by Peixoto et al. [13] for cherry stems. On the other hand, the different geographical origin of lemon verbena from the commercial brand A might be another reason that had an impact on the low antioxidant activity and bioactive compounds contents. Although Portugal and Spain are geographically very close and have similar climates, there are different regions, even within the same country, which have more adverse weather conditions than others [4,19]. Thus, some factors, such as the cultivation and harvest seasons, the type of soil, and the conditions to which this soil was subjected, might have markedly influenced the amount and the type of bioactive components produced by lemon verbena of brand A and, consequently, the results herein obtained. In fact, it is important to emphasize that these compounds, particularly phenolics, are secondary metabolites normally produced by plants as a defense mechanism against external agents and, therefore, their production is very dependent on the type of environment to which the plant is subjected [4,16,20]. The phenolic characterization of all infusions by UHPLC-ESI-QTOF-MS proved to be an important tool to ascertain which of the two factors herein described might have had more impact on the significantly low results of lemon verbena infusions from brand A, as will be further discussed.

Regarding the results obtained for lemon verbena infusions of the remaining commercial brands (B, C, and D), quite similar results were found between infusions from brands B and D (except in ORAC assay). Moreover, infusions from brand C presented the highest results in almost all assays (except in DPPH^•^ scavenging and ORAC), and, for FRAP and Folin–Ciocalteu assays, the results of these infusions were significantly higher than those obtained for brands B and D infusions (Table 1). Contrariwise, brand B samples presented the highest antiradical activity in both DPPH^•^ scavenging and ORAC assays. These different results are related to the mechanisms of action implicated in each assay and with the different proportion of compounds probably existent in each commercial brand, which might interact with each other in several ways, leading to different antioxidant actions. Nevertheless, according to the Pearson correlations achieved with the results of the different assays (Table 2), it is possible to observe high correlations between all assays. Thus, it can be suggested that phenolic compounds (including flavonoids) are the main ones responsible for the antioxidant activities of lemon verbena infusions by all mechanisms herein addressed (electron transfer and H atom transfer mechanisms). More specifically, although high correlations at a significant level of 0.01 have been found between all assays, the highest correlations were found between the FRAP assay and the total contents of phenolic and flavonoid compounds, which suggests that these compounds were the main ones responsible for the electron transfer mechanisms ascertained by the FRAP assay. The slightly lower (but also significant) correlations found between the total contents of phenolic and flavonoid compounds and ORAC assay might be possibly explained by the existence of other compounds rather than phenolics that might have also exhibited the capacity to neutralize peroxyl radicals by H atom transfer mechanisms. Notwithstanding, it is evident that phenolics were also the principal compounds responsible for the H atom transfer mechanisms, even because DPPH^•^ scavenging assay (where the DPPH^•^ radical might be neutralized by both electron transfer or H atom transfer mechanisms) was similarly correlated with both FRAP and ORAC assays (where the different mechanisms are individually evaluated in each assay) and also with phenolic and flavonoid contents.

### 3.2. Chemical Characterization by UHPLC-ESI-QTOF-MS

After the assessment of the antioxidant activity and the total contents of phenolic and flavonoid compounds in commercial lemon verbena infusions by different spectrophotometric assays, comprehensive phenolic profiling by UHPLC-ESI-QTOF-MS/MS was accomplished to better characterize the infusions studied in this work, as well as to explain the observed antioxidant activity.

**Figure 1:**
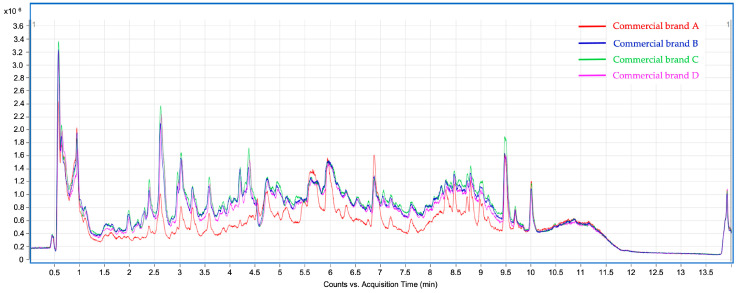
Total Ion Chromatograms (TIC) corresponding to the phenolic profile of lemon verbena infusions from commercial brands A, B, C, and D were analyzed by UHPLC-ESI-Q-TOF-MS/MS.

At first sight, the chromatographic profiles obtained for the studied infusions exhibit clear differences as well as similarities, in line with the results obtained in the previous assays. The total ion chromatograms (TIC) of the analyzed infusions (Figure 1) show significant differences between commercial brand A and the remaining brands (B, C, and D). Despite their qualitative similarities, the TIC intensity of brand A was significantly lower than the others, thus suggesting a poorer phenolic content. This assumption was confirmed because most of the thirty-four tentatively identified compounds exhibit significantly lower peak areas in brand A infusions, as discussed below. Table 3 summarizes the tentatively identified compounds according to their respective retention time (min), experimental and theoretical *m*/*z* for deprotonated molecular ion ([M–H]^−^), calculated mass error (ppm), MS/MS product ions, and molecular formula. These phytochemicals are classified into six different groups of compounds (hydroxybenzoic acids, hydroxycinnamic acids, flavonols, flavones, phenylethanoids, and iridoids). Compounds **16**, **19**, **21**, **23**, **24**, and **27**–**29** are hydroxycinnamic acids conjugated to a phenylethanoid moiety, as will be further described. Hence, for comparative purposes, these eight compounds were classified as a subgroup within the hydroxycinnamic acids group, different from the free hydroxycinnamic acid derivatives. Table 4 shows the relative abundances, in terms of chromatographic peak area, for the compounds identified, as well as the abundance percentages for the different classes and subclasses of compounds. Chromatographic peak areas were obtained from the high-resolution extracted ion chromatograms (HREIC) using the deprotonated molecular ion ([M–H]^−^).

Regarding the chemical characterization of the samples, thirty-four phenolic compounds were tentatively identified. The iridoids class, well-described for plants from the genus *Lippia* [8,23,24] and widely known as one of the main classes of compounds found in lemon verbena [2,7,8,21], is well-represented in the analyzed infusions. A total of six iridoid compounds (**2**–**6** and **13**) were tentatively identified, some of them with moderately intense chromatographic peak areas. Compounds **2** and **6** were tentatively identified as two isomers of gardoside, exhibiting the same deprotonated molecular ion ([M–H]^−^ at *m*/*z* 373) and quite similar MS-MS spectra. The fragment ion at *m*/*z* 211 (found only in compound **6**) resulted from the loss of a hexose moiety (162 u) and subsequent loss of water ([M–H–glucose–18]^−^) and CO_2_ ([M–H–glucose–44]^−^), leading two fragment ions at *m*/*z* 193 and 167. Moreover, fragment ions at *m*/*z* 149 [M–H–glucose–H_2_O–CO_2_]^−^ and *m*/*z* 123 [M–H–glucose–COOHCH_2_CHO]^−^ were also found in both isomers. These fragment ions are characteristic of gardoside, as well as of other iridoid glycosides [2,8].

Compounds **4** and **13**, tentatively identified as shanzhiside and theveside, respectively, presented fragmentation patterns quite similar to gardoside. In the case of shanzhiside, fragments detected at *m*/*z* 229, 211, and 185 correspond to [M–H–glucose]^−^, [M–H–glucose–H_2_O]^−^, and [M–H–glucose–CO_2_]^−^ ions, respectively. Other shanzhiside fragments at *m*/*z* 167 [211–CO_2_–H_2_O]^−^, *m*/*z* 149 [229–CO_2_–H_2_O]^−^, and *m*/*z* 123 [229–COOHCH_2_CHO–H_2_O]^−^ were also found. Theveside exhibited *m*/*z* 209 and 121 ions corresponding to [M–H–glucose]^−^ and [M–H–glucose–COOHCH_2_CHO]^−^ fragments. In addition, the deprotonated molecular ion of theveside undergoes decarboxylation, resulting in a fragment at *m*/*z* 345 [M–H–CO_2_]^−^. Successive losses of a hexose moiety and a water molecule led to the fragments at *m*/*z* 183 [M–H–CO_2_–glucose]^−^ and *m/z* 165 ([M–H–CO_2_–glucose–H_2_O]^−^). Both shanzhiside and theveside presented a fragment ion at *m/z* 101, corresponding to a methyl 3-oxopropanoate molecule. This fragment is common to iridoid compounds containing a typical hemiacetal group, which can easily form an epimeric isomer, being isomerized to two aldehyde groups. Consequently, the C-5 and C-6 positions of the isomer form a double bond, and the methyl 3-oxopropanoate is lost [9].

Compound **5**, which was tentatively identified as ixoside, also shows *m*/*z* 101 fragment ion in the MS/MS spectrum, as well as other characteristic fragments from iridoid glycosides. As observed in the theveside fragmentation pattern, the deprotonated molecular ion of ixoside also undergoes decarboxylation, resulting in the *m*/*z* 343 fragments. The base peak at *m*/*z* 181 is generated after the removal of a glucose moiety. Further fragmentation of the base peak leads to *m*/*z* 163 [181–H_2_O]^−^, *m*/*z* 137 [181–CO_2_]^−^, and *m*/*z* 119 [181–CO_2_–H_2_O]^−^ product ions.

**Figure 2:**
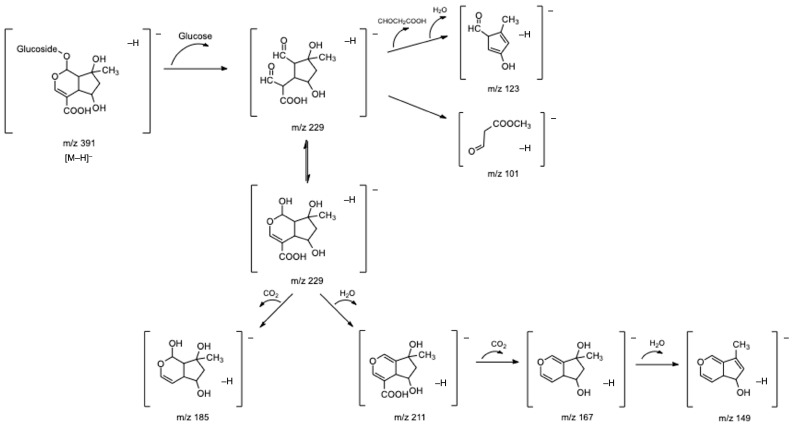
Fragmentation pathway proposed for shanzhiside.

The five iridoid glycosides described above have already been described in the literature for lemon verbena. However, a gardoside derivative (compound **3**) was, as far as we know, identified for the first time in our study in all commercial infusions. This compound presented a base peak at *m*/*z* 373, which resulted from the neutral loss of a methylglucuronide moiety (208 u) and which corresponded to the deprotonated molecular ion of gardoside. Furthermore, the MS-MS spectrum of this compound also revealed fragment ions coincident with those found for gardoside isomers and, therefore, was tentatively identified as gardoside-*O*-methylglucuronide. Figure 2 presents a proposed fragmentation pathway of shanzhiside as an example of the fragmentation pattern of iridoid glycosides.

Regarding hydroxybenzoic acids, this class of compounds—although quite prevalent in plants and well-described for many of them [25,26]—is being described, as far as we know, for the first time in infusions of commercial lemon verbena. In this study, three glycosylated derivatives of hydroxybenzoic acids (compounds **1**, **7**, and **9**) were detected and tentatively identified based on the data found in databases. All of them presented in their MS-MS spectra fragment ions resultant from the loss of a glucose moiety (162 u) (at *m*/*z* 153 in compound **1** and at *m/z* 137 in compounds **7** and **9**), corresponding to their respective aglycones (dihydroxybenzoic acid in compound **1**, and hydroxybenzoic acid in compounds **7** and **9**). Moreover, compounds **1** and **9** also presented fragments at *m*/*z* 109 (151–CO_2_]^−^) and 93 (137–CO_2_]^−^), respectively, which are characteristic of these aglycones, thus helping to elucidate the identification of compounds. Although these three compounds have been detected in residual amounts in our infusions, their detection might be of extreme importance once this class has some bioactive properties well-recognized, namely antioxidant, anti-inflammatory, anti-diabetic, antimicrobial, antifungal, chemopreventive, and neuroprotective [27,28]. Thus, these compounds might contribute to the biological properties of this plant by additively or synergistically interacting with other compounds present in lemon verbena.

**Figure 3:**
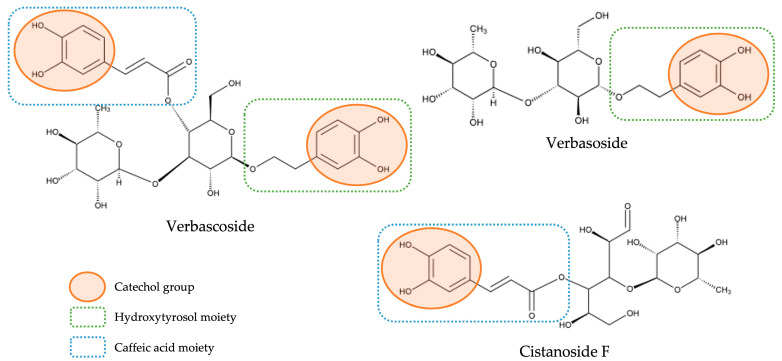
Chemical structures of verbascoside, verbasoside, and cistanoside F and their structure-antioxidant activity relationships.

With respect to the huge class of hydroxycinnamic acids—which represented the main class of compounds both in qualitative and quantitative terms—as previously mentioned, the compounds identified were subdivided into two distinct groups: a group constituted only by hydroxycinnamic acids not containing a phenylethanoid moiety (here denominated as free hydroxycinnamic acids) and a group constituted by hybrid compounds containing a hydroxycinnamic acid moiety and a phenylethanoid moiety (here designated as hydroxycinnamic acid conjugated to phenylethanoid). We have decided to differentiate them not only because the second ones stood out in quantitative terms, being already widely acknowledged as the main bioactive compounds present in lemon verbena [7,17,21], but also because these hybrid compounds, due to their distinct molecular characteristics, may present structural–activity relationships different from the observed for compounds that do not contain the phenylethanoid portion. In fact, in a recent study with lemon verbena extracts, the importance of the structure of the compounds in their antioxidant power was demonstrated and ascertained by different mechanisms of action [17]. For example, the antioxidant activity obtained in that study for verbascoside (which contains a caffeic acid moiety and a hydroxytyrosol moiety) was much higher than verbasoside (which contains only a hydroxytyrosol moiety), and this, in turn, was higher than cistanoside F (which contains only a caffeic acid moiety) and, according to this study, this can be explained by their structure. First, it was confirmed that catechol moiety existing in phenolic compounds is the main responsible for the antioxidant activity of these compounds, as had already previously described in other studies [29,30]. Thus, this explains the strongest antioxidant activity of verbascoside compared with the other two compounds once, as can be observed in Figure 3, which presents the chemical structures of these three compounds; verbascoside contains two catechol moieties, while verbasoside and cistanoside F have only one. Moreover, the higher antioxidant activity observed for verbasoside when compared with cistanoside F was explained due to the different class of compounds to which each one belongs; although both contain one catechol group, verbasoside belongs to the phenylethanoid class (being derived from hydroxytyrosol), while cistanoside F belong to the hydroxycinnamic acid class (being derived from caffeic acid) and it is already described that hydroxytyrosol has a stronger antioxidant activity than caffeic acid [17]. In this way, it is important to approach these different compounds as belonging to independent groups with independent characteristics.

Concerning free hydroxycinnamic acids, five compounds strictly related to each other (compounds **10**–**12**, **14**, and **17**) have been tentatively identified and, to the best of our knowledge, only two (compounds **10** and **17**) had already been described for *Lippia citriodora*. Compound **10** was the major free hydroxycinnamic acid detected in our infusions and was tentatively identified as cistanoside F due to the characteristic base peak at *m*/*z* 179 corresponding to caffeic acid. Moreover, besides cistanoside F, its proper biosynthetic precursor—caffeic acid (compound **14**)—as well as two hexosides of caffeic acid (compounds **11** and **12**) were detected in our infusions and, as far as we know, are being described for the first time in infusions of lemon verbena, contrarily to cistanoside F, which is already well-documented for this plant [2,8]. Caffeic acid was tentatively identified taking into account the molecular formula provided, the characteristic fragment ion at *m*/*z* 135 ([M–H–CO_2_]^−^), and the similarity found between its retention time and the one previously found in our laboratory, applying the same analytical workflow [13]. The tentative identification of compounds **11** and **12** were made following the same line of thought: the loss of a hexose moiety (162 u) from the [M–H]^−^, resulting in the fragment at *m*/*z* 179 (caffeic acid), together with its fragment at *m*/*z* 135, allowed their tentative identification as caffeic acid hexosides. Moreover, the fragment at *m*/*z* 161 [M–H–H_2_O]^–^, also characteristic of caffeic acid MS-MS spectra, as well as fragment ions at *m*/*z* 281, 251, and 221—resultant from neutral losses of portions of the hexoside—were also detected, thus enhancing our tentative identification. Although caffeic acid and its hexoside derivatives are being described for the first time in lemon verbena infusions, their presence in this plant was already presupposed because caffeic acid is synthesized by all plant species, where it might be found in innumerous forms, namely in glycosidic forms, and it is the base for the biosynthesis of several compounds, such as monomers (e.g., cistanoside F), organic acid esters (e.g., verbascoside and its analogous), dimers, trimers, or flavonoids [30]. Caffeic acid itself might be a degradation product of its derivatives, i.e., it might have resulted from the cleavage of cistanoside F, verbascoside, or other caffeic acid derivatives here detected. The last free hydroxycinnamic acid detected in lemon verbena infusions (compound **17**) was tentatively identified as *p*-coumaric acid, and it is also highly related to caffeic acid because it is its immediate biosynthetic precursor in the shikimate pathway [30]. This compound was identified according to the literature and to MS-MS databases, where the fragment at *m*/*z* 163 ([M–H–CO_2_]^−^), found in our MS-MS spectrum, is also well-described [22].

**Figure 4:**
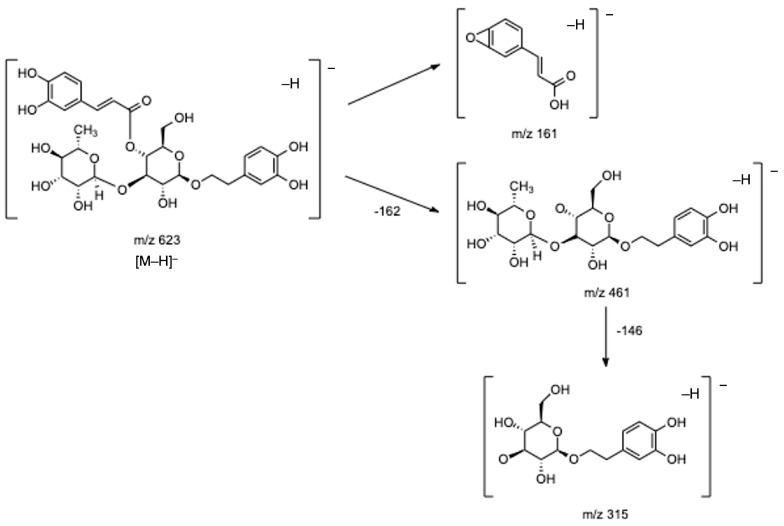
Fragmentation pathway proposed for verbascoside.

In respect of hydroxycinnamic acid conjugated to phenylethanoid subgroup, the most important group identified in lemon verbena [1,7,8,17,21], and particularly in our infusions, eight compounds (**16**, **19**, **21**, **23**, **24**, and **27**–**29**) have been tentatively identified. Of these, compounds **21** and **23** were the main ones detected in lemon verbena infusions, and based on their fragmentation patterns and on data previously reported, they might correspond to verbascoside or its isomers isoverbascoside and forsythoside A [2,5,7,8,21,22]. Both compounds presented the base peak at *m*/*z* 623 ([M–H]^−^) and three fragments at *m*/*z* 461 [M–H–caffeoyl]^−^, *m*/*z* 315 [461–rhamnose]^−^, and *m*/*z* 161 [caffeic acid–H–H_2_O]^−^. Figure 4 presents the fragmentation pattern of verbascoside as an example. Although it was not possible to confirm the identity of the isomers, as previously mentioned, verbascoside is well-described as the main compound found in lemon verbena [1,2,7,22], being in several studies acknowledged as the main one responsible for the several biological properties mentioned in the Introduction section. Specifically, regarding the antioxidant activity, Sanchez-Marzo et al. showed that verbascoside presented the strongest antioxidant activity of all compounds detected in lemon verbena extracts, being inclusive higher than its two isomers isoverbascoside and forsythoside A, which was explained due to the position of caffeoyl moiety, reflecting the important relationship between structure and antioxidant activity [17] again. Notwithstanding, it is important to highlight that according to some studies, the antioxidant activity of whole lemon verbena extracts is higher than isolated verbascoside extract because the compounds present in the whole plant extract, even when in lower amounts, might interact with each other in possibly additive, synergistic, or antagonistic ways, potentiating the effects of verbascoside [6,7,8].

Compounds **16** and **19** presented the same [M–H]^−^ and similar MS-MS spectra and have been reported in the literature as β-hydroxylated derivatives of verbascoside and isoverbascoside [7,8,21]. Both MS-MS spectra contained fragment ions at *m*/*z* 621 [M–H–H_2_O]^−^, 459 [M–H–caffeic acid]^−^, 179 [caffeic acid–H]^−^, and 161 [caffeic acid–H–H_2_O]^−^.

The four remaining hybrid compounds (**24**, **27**–**29**), instead of a caffeoyl portion, contained a feruloyl one and were easily detected by the characteristic release of this moiety (at *m*/*z* 193) from the respective deprotonated molecular ion. Compounds **24** and **27** were identified as eukovoside isomers (I and II) because they presented the same [M–H]^−^, at *m*/*z* 637, and similar fragmentation patterns, which also corroborated with those described in the literature [2,7,8,21,22]. The loss of the feruloyl moiety resulted in the characteristic fragment ion at *m*/*z* 461, corresponding to the phenylethanoid group attached to rhamonopyranosyl-glucopyranoside, and after which resulted in the fragment at *m*/*z* 315 [461–rhamnose]^−^. A fragment at *m*/*z* 175 [ferulic acid–H–H_2_O]^−^ was also found. In turn, compounds **28** and **29** also presented the same deprotonated molecular ions but at *m*/*z* 651, being tentatively identified as isomers of martynoside, which was also based on data reported in the literature [2,8,21,22]. As compounds **24** and **27**, martynoside isomers also presented fragments resultant from losses of feruloyl and rhamnosyl moieties, as well as a fragment at *m*/*z* 591 resultant from neutral loss of a portion of the glucopyranoside moiety. The fact that these four compounds contain a feruloyl moiety rather than a caffeoyl moiety in their structure makes them less potent antioxidant compounds than verbascoside and its derivatives because ferulic acid instead contains two hydroxyl groups *ortho* to each other in its aromatic ring (forming the catechol group which, as it was previously mentioned, is the main one responsible for the greater antioxidant activity of strong phenolic compounds, and is present in caffeic acid) and contains a methoxy group and a hydroxyl group *ortho* to each other, thus decreasing, for example, the efficiency of the scavenging reaction of peroxyl radicals [17,31]. Furthermore, it might be expected that martynoside has a lower antioxidant activity than eukovoside since, aside from the absence of a catechol group in the hydroxycinnamic acid moiety of martynoside, there is also the absence of a catechol group in the phenylethanoid moiety due to the existence of another methoxy group in the aromatic ring instead of a hydroxyl group.

Concerning the phenylethanoids class, as already mentioned several times throughout the text (although it appears that only compound **8** is part of this class), it is, in truth, composed of a total of nine compounds (compound **8**—which is a phenylethanoid not conjugated to any other class of compounds—and compounds **16**, **19**, **21**, **23**, **24**, and **27**–**29**, which are already described above and belong to both hydroxycinnamic acids and phenylethanoids classes). Summing the percentages of conjugated and non-conjugated phenylethanoids, it is possible to see the prominence of this class in lemon verbena, which is mainly attributed to verbascoside and its derivatives. The only non-conjugated phenylethanoid detected here (compound **8**) has also demonstrated to be a relevant compound in lemon verbena infusions because it also presented moderately high chromatographic peak areas in all brands, and it was tentatively identified as verbasoside by comparison of our MS-MS spectrum with those reported in previous works [2,8,22]. Its MS-MS spectrum presented the [M–H]^−^ at *m*/*z* 461 as the base peak, which then successively lost a rhamnose moiety and a water molecule, resulting in fragments at *m*/*z* 315, and 297, respectively. Moreover, fragments at *m*/*z* 161 [hexose–H–H_2_O]^−^, 135 [hydroxytyrosol–H–H_2_O]^−^, and 113 [hexose–H–H_2_O–CH_4_O_2_]^−^ were also detected.

Finally, the large family of flavonoids constituted the second main class of compounds present in lemon verbena, both in qualitative and quantitative terms, with eleven compounds identified in total, despite all of them belonging to only two subclasses: flavonols (compound **22**) and flavones (compounds **15**, **18**, **20**, **25**, **26**, **30**–**34**).

The only flavonol herein detected (compound **22**) was present in all commercial brands in residual amounts and was tentatively identified as isorhamnetin-7-*O*-glucuronide based on the detection of fragments resultant from successive neutral losses of a glucuronide moiety and a methyl group, also detected in the literature and databases.

Regarding flavones subclass, four diglucuronide derivatives of four different flavones were detected: luteolin-7-diglucuronide (compound **15**), apigenin-7-diglucuronide (compound **18**), chrysoeriol-7-diglucuronide (compound **20**), and acacetin-7-diglucuronide (compound **26**). All of these compounds present in their MS-MS spectra, a fragment ion at *m*/*z* 351 resulted from the loss of the respective aglycone of the compound, and a fragment ion at *m*/*z* 193, corresponding to [glucuronic acid–H]^−^. Furthermore, for compounds **15** and **18**, fragments were also observed at *m*/*z* 285 [luteolin–H]^–^ and 269 [apigenin–H]^−^, respectively, and for compounds **18** and **26**, a fragmentation product of glucuronic acid (at *m*/*z* 113) was additionally found. The fragment at *m*/*z* 461 detected in compound **26** was consistent with [acacetin–H–glucuronide]^−^ ions.

The remaining six flavones detected in this study (compounds **25**, **30**–**34**) correspond to methoxyflavones derivatives, whose fragments mainly resulted from successive neutral losses of methyl groups. Compound **25** was tentatively identified as trihydroxymethoxyflavone glucuronide, and, as far as we know, it had not yet been described for infusions and/or other extracts from this plant. The loss of a glucuronide moiety (176 u) from the [M–H]^−^ resulted in an aglycone at *m*/*z* 299, which might correspond to various trihydroxymethoxyflavones, namely to any of the molecules tentatively identified for compound **31** (hispidulin, diosmetin, and chrysoeriol), all previously described for lemon verbena. Beyond that, the fragment at *m*/*z* 284 [trihydroxymethoxyflavone–CH_3_]^–^ and the fragments at *m*/*z* 113 and 175 (two fragmentation products of glucuronic acid) also aided in tentatively identifying this new compound. The remaining methoxyflavones derivatives were already described in the literature for this plant, as well as the other four flavones [1,2,3,7,8,21,22]. Of all flavonoids detected, luteolin-7-diglucuronide (compound **15**) stood out in quantitative terms, representing the main flavonoid detected in lemon verbena infusions and the third compound overall with the highest chromatographic peak areas (behind compounds **21** and **23**), which is in accordance with previous reports about lemon verbena [3,22].

The heat map in Table 4 highlights the different concentrations of the compounds in the analyzed samples, using a color code ranging from dark to light color, being possible for each compound individually to understand if it was present in higher or lower amounts in the different infusions. On the basis of these data, some assumptions can be made to explain the impact of the lack of package and the geographical origin on the composition of the different commercial infusions. The compounds described in this study were found in the four commercial brands, with the exception of compound **17** (*p*-coumaric acid), which was not detected in brand C infusions. However, target compounds were found in different proportions in the analyzed samples. In terms of abundance percentage, infusions from brand A presented a global percentage of flavonoids significantly higher than the other samples, while the percentages of phenylethanoids and iridoids were slightly lower. However, for brands B, C, and D, the percentage values obtained for the different classes of compounds were quite similar to each other. Hence, it seems that the different geographical origin of brand A has an impact on the phenolic content of its infusions. In fact, there are several studies comparing the phenolic profiles and the bioactive properties of plants from different geographical origins, showing that, despite the qualitative profile being quite similar for a plant species from different origins, the quantitative profile and, consequently, the bioactive properties can be quite distinct [32,33,34]. As already mentioned, these differences can eventually be explained due to different biotic and abiotic factors that might influence the chemical composition of the plant and, consequently, its biological potential [4,33,34]. The hypothesis of the lack of packaging of brand A samples cannot, however, be ruled out, although we consider that it is less likely to have been the cause for the significant differences observed because the class of flavonoids is described to be quite susceptible to external factors, namely to light, air, and temperature [35,36].

Furthermore, the detection and tentative identification of eight compounds that were not yet described for lemon verbena infusions and/or other extracts was also quite interesting and may have different justifications. First, considering that the plant material used in this study derives from industrially produced herbal preparations, it is possible that the commercial samples were contaminated with other plants containing these compounds. Nonetheless, considering that all these new compounds were detected in all the commercial infusions studied from the different selected brands (and the phenolic profile was qualitatively similar for all samples), this hypothesis seems very unlikely. Second, the analytical technique used in the present study (UHPLC-ESI-QTOF-MS, operating in high-resolution tandem-mass spectrometry mode (HRMS/MS)) is a valuable tool to detect and identify unknown compounds as it provides high-quality MS data for reliable identification after convenient application of systematic HRMS/MS data-mining strategies (mass defect filtering, diagnostic fragment ion filtering, background subtraction filtering, or neutral loss filtering). Thus, it allows the identification of new compounds with high accuracy. In addition, considering that some of these new compounds are structurally related to others already described (e.g., aglycones, glucosides, and/or moieties of other identified compounds), it might also be possible that some of these compounds have resulted from the extraction process where the high temperatures achieved, for example, might have degraded/cleaved/changed the structure of other related compounds present in the original samples.

In sum, further studies with a higher number of samples duly authenticated and with their origin, production, processing, storage, and other important conditions very well-described would be needed to deeply explore these results and hypotheses. Particularly, as it was not the scope of this work, it would be interesting in future studies to not use commercial samples but samples that are grown in a laboratory to verify whether these compounds appear in fresh and dry plants and whether there is any influence of the extractive process, including temperature, on the appearance of these compounds.

### 3.3. Free Amino Acid Profile by RP-HPLC-FLD

Table 5 shows the free amino acid profile of lemon verbena infusions. The amino acids in higher concentrations were aspartic acid (Asp), glutamic acid (Glu), asparagine (Asn), and proline (Pro). In fact, it is already described that Asp and Glu are the most abundant amino acids in plants, which is in accordance with our results [12]. The remaining 12 amino acids detected were generally found in moderate amounts, which denotes the richness of lemon verbena in free amino acids. Indeed, comparing this profile with the one obtained in our previous study with rosemary infusions [18], where only seven amino acids were detected, lemon verbena showed to be a better source of several free amino acids. These findings are of extreme importance since, besides the physiological roles already well-recognized, some amino acids may also be endowed with antioxidant properties [11,12,37], thus enhancing the antioxidant potential of this plant. To ascertain this potential in lemon verbena infusions, we have determined the Pearson correlations between the contents of free amino acids detected in all commercial brands and the results obtained in the spectrophotometric assays (Table 6). Of all amino acids, phenylalanine (Phe) was the one that presented more significant correlations with the antioxidant activity results, mainly with DPPH^•^ scavenging ability (0.992; *p* < 0.01) and FRAP values (0.969; *p* < 0.05). In fact, Guidea et al. [11] had already documented the high capacity of this amino acid to scavenge not only DPPH^•^ radicals but also superoxide radicals. The correlations between Phe content and FRAP values might be explained due to the presence of an amino group attached to a phenyl moiety in its structure which, therefore, favors the electron transfer mechanisms that are determined in the FRAP assay. Although Phe has been the only amino acid whose correlations with antioxidant activity assays were more significant, it should be noted that amino acids may act by other mechanisms of action rather than those ascertained in our study. For example, Asp and Glu have already proven to be helpful in decreasing oxidative stress because they present high free radical quenching activity against various radicals, namely a high ability to protect myoglobin against peroxynitrite radicals [11,38]. In addition, Asn is described to possess the highest capacity to scavenge superoxide radicals and to chelate metal ions when compared with the other amino acids, and also to have a high capacity to protect myoglobin against peroxynitrite radicals, such as Asp and Glu [11,38]. Therefore, although they had not presented significative results in our study, these and some other amino acids may contribute to the overall antioxidant activity of lemon verbena infusions, but by other mechanisms not herein determined.

Regarding the concentrations of free amino acids obtained from each commercial brand, in general, no significant differences were noticed between samples, with some exceptions. Tyrosine (Tyr) and hydroxyproline (Hyp) were only detected in brand C infusions, although in very low amounts. Moreover, Phe was present in significantly lower amounts in brand A compared with the others. This finding is quite curious because Phe is a precursor of the huge variety of phenolic compounds, including the ones identified in our study [39,40,41], which were equally present in significantly lower amounts in brand A infusions. In fact, correlations were found at a significant level (*p* < 0.05) between the contents of phenylalanine and the contents of total phenolic and flavonoid compounds (Table 6). Therefore, as Phe was present in low amounts in lemon verbena from brand A, the synthesis of phenolic compounds in this cultivar might have occurred to a lower extent when compared with the others, thus explaining the low results achieved for infusions from this brand. Additionally, although additional studies should be needed, the low amounts of Phe, which resulted in the low amounts of phenolic compounds, might be explained, once again, due to the distinctive geographic origin of samples from commercial brand A, enhancing the aforementioned hypothesis even more. In a previous study, it was found that tobacco plants grown in a region with a drastic climate (high temperature and low rainfall) produced higher levels of Phe and its respective defense-related metabolites (resultant from shikimate-phenylpropanoid metabolism) than tobacco plants grown in a region with more a moderate climate due to their need to protect themselves against oxidative stress caused by environmental growth conditions [41]. Thus, in the case of lemon verbena, the samples from commercial brand A (originated from Spain) might have been subjected to milder climate conditions than the samples from commercial brand B, C, and D infusions (all originated from Portugal), resulting in the lower contents in Phe and phenolics. Finally, concerning the aromatic amino acids (Tyr, Trp, and Phe), which are all produced from chorismate (the final product of the shikimate pathway) [39,40,42], the fact that Trp has not been detected in any sample and that Tyr has been found only in brand C infusions and in very low concentrations might be explained by the fact that the synthesis of these two amino acids in plants must be strictly regulated to ensure that the highest carbon flux is directed to Phe [39]. The higher requirements of Phe rather than Tyr and Trp are because phenylalanine-derived compounds (including phenolic compounds) may represent around 30–45% of organic matter in plants, whereas tyrosine derivatives (e.g., betalains, isoquinoline alkaloids) and tryptophan derivatives (e.g., indole alkaloids, phytoalexins, auxin) are not needed or present in such amounts [39].

## 4. Conclusions

The present study allowed us to achieve some insights into lemon verbena infusions from different commercial brands. First, as expected, their antioxidant potential was demonstrated by the various methodologies implemented, as well as the importance of phenolic compounds (including flavonoids) in this potential because they were shown to be the main compounds responsible for the antioxidant activity of these infusions. Furthermore, the geographical origin of the different samples (acquired from different commercial brands) seemed to have a great influence on the antioxidant activity and on their composition in phenolic compounds and also in some amino acids, particularly in phenylalanine, which is the precursor of these secondary metabolites, although a study with a higher number of samples properly authenticated would be needed to confirm this hypothesis with confidence.

The UHPLC-ESI-QTOF-MS analysis allowed us to achieve a better characterization of the different lemon verbena infusions and to explain the results primarily obtained in the spectrophotometric assays. In total, thirty-four compounds were tentatively identified in all infusions, of which verbascoside and its analogs, as well as luteolin-7-diglucuronide—both described for lemon verbena and with well-known antioxidant properties—have stood out in semi-quantitative terms. The detailed analysis by UHPLC-ESI-QTOF-MS allowed us to tentatively identify eight compounds that are not yet described in the literature, to the best of our knowledge, in lemon verbena infusions (dihydroxybenzoic acid glucoside, gardoside-*O*-methylglucuronide, two isomers of hydroxybenzoic acid-*O*-glucoside, two isomers of caffeic acid-*O*-glucoside, caffeic acid, and trihydroxymethoxyflavone glucuronide). Finally, in this study, the analysis of the free amino acid profile of lemon verbena infusions was also conducted, as far as we know, for the first time. The commercial lemon verbena infusions showed to be a good source of free amino acids, some of which with recognized antioxidant activities. Considering that the plant material analyzed in this study derives from industrially produced herbal preparations, with many unknown aspects that could impair the authenticity of the samples (e.g., possible contamination with other plant species, unspecified exact geographical origin, undefined production, processing, and storage conditions to which samples were subjected, etc.), it will be essential in future studies to include, for example, DNA/PCR techniques to prove the samples’ authenticity. Even so, the present study highlights the antioxidant potential of lemon verbena infusions easily accessible to consumers and its richness in compounds with relevant bioactive properties, showing that at least the consumers of these commercial lemon verbena infusions may obtain good sources of antioxidants and amino acids in their diets.

## Figures and Tables

**Table 1:** Mean values (±standard deviation) of the levels of antioxidant activity and bioactive compounds for lemon verbena infusions prepared from different commercial brands.

Commercial Brand	Antioxidant Activity			Bioactive Compounds	
	DPPH^•^ Inhibition (μg TE/mL)	FRAP (μg FSE/mL)	ORAC (μg TE/mL)	Total Phenolic Content (μg GAE/mL)	Total Flavonoids Content (μg CE/mL)
**A**	23.07 ± 1.89 ^b^	1030.50 ± 123.74 ^c^	300.10 ± 4.97 ^c^	59.72 ± 4.44 ^c^	55.42 ± 0.00 ^b^
**B**	67.40 ± 1.48 ^a^	3805.00 ± 35.36 ^b^	1022.59 ± 20.90 ^a^	227.22 ± 5.56 ^b^	210.28 ± 18.03 ^a^
**C**	66.34 ± 1.11 ^a^	4755.00 ± 176.78 ^a^	853.21 ± 19.54 ^ab^	269.44 ± 8.89 ^a^	254.72 ± 21.40 ^a^
**D**	67.18 ± 0.92 ^a^	3563.33 ± 381.88 ^b^	707.03 ± 66.80 ^b^	227.78 ± 7.22 ^b^	215.14 ± 19.34 ^a^

Within each column, different letters represent significant differences between mean values at *p* < 0.05.

**Table 2:** Pearson correlations were obtained with the results of different assays.

	DPPH^•^ Inhibition	FRAP	ORAC	Total Phenolic Content	Total Flavonoids Content
DPPH^•^ Inhibition	1	0.949 *	0.923 *	0.970 *	0.966 *
FRAP	0.949 *	1	0.879 *	0.990 *	0.983 *
ORAC	0.923 *	0.879 *	1	0.895 *	0.883 *
Total Phenolic Content	0.970 *	0.990 *	0.895 *	1	0.998 *
Total Flavonoids Content	0.966 *	0.983 *	0.883 *	0.998 *	1

* Correlation is significant at the 0.01 level (two-tailed).

**Table 3:** Tentative identification of compounds detected in lemon verbena infusions by UHPLC-ESI-QTOF-MS.

Peak	Retention Time (min)	[M–H]^−^ Experimental	[M–H]^−^ Theoretical	Error (ppm)	MS^2^ Productions	Molecular Formula	Family	Tentative Identification	Refs.
1	2.111	315.0722	315.0722	−0.13	108 (68), 109 (42), 152 (100), 153 (53)	C_13_H_16_O_9_	Hydroxybenzoic acid	Dihydroxybenzoic acid glucoside	
2	2.298	373.1144	373.1140	−1.01	123 (81), 149 (75), 167 (53), 193 (100)	C_16_H_22_O_10_	Iridoid	Gardoside (I)	[2,8,21]
3	2.358	581.1730	581.1723	−1.16	149 (10), 167 (9), 211 (13), 373 (100)	C_23_H_34_O_17_	Iridoid	Gardoside-*O*-methylglucuronide	
4	2.391	391.1256	391.1246	−2.59	101 (21), 119 (32), 123 (57), 149 (30), 167 (100), 185 (24), 211 (15), 229 (28)	C_16_H_24_O_11_	Iridoid	Shanzhiside	[2,21]
5	2.605	387.0938	387.0933	−1.32	101 (45), 119 (64), 137 (79), 163 (64), 181 (100), 343 (29)	C_16_H_20_O_11_	Iridoid	Ixoside	[2]
6	2.638	373.1148	373.1140	−2.08	123 (100), 149 (87), 167 (24), 193 (7) 211 (18)	C_16_H_22_O_10_	Iridoid	Gardoside (II)	[2,8,21]
7	2.885	299.0779	299.0772	−2.20	137 (100)	C_13_H_16_O_8_	Hydroxybenzoic acid	Hydroxybenzoic acid-*O*-glucoside (I)	
8	3.028	461.1680	461.1665	−3.35	113 (45), 135 (28), 161 (12), 297 (5), 315 (10), 461 (100)	C_20_H_30_O_12_	Phenylethanoid	Verbasoside	[2,8,22]
9	3.158	299.0773	299.0772	−0.19	93 (59), 137 (100)	C_13_H_16_O_8_	Hydroxybenzoic acid	Hydroxybenzoic acid-*O*-glucoside (II)	
10	3.255	487.1464	487.1457	−1.40	179 (100)	C_21_H_28_O_13_	Free hydroxycinnamic acid	Cistanoside F	[2,8,21]
11	3.318	341.0884	341.0878	−1.69	135 (42), 161 (47), 179 (100), 221 (30), 251 (13), 281 (34)	C_15_H_18_O_9_	Free hydroxycinnamic acid	Caffeic acid-*O*-hexoside (I)	
12	3.495	341.0887	341.0878	−2.57	135 (47), 161 (31), 179 (100), 221 (50), 251 (22), 281 (30)	C_15_H_18_O_9_	Free hydroxycinnamic acid	Caffeic acid-*O*-hexoside (II)	
13	3.588	389.1102	389.1089	−3.24	101 (50), 121 (100), 165 (53), 183 (24), 209 (26), 345 (29)	C_16_H_22_O_11_	Iridoid	Theveside	[2,8,21]
14	3.858	179.0345	179.0350	2.70	135 (100)	C_9_H_8_O_4_	Free hydroxycinnamic acid	Caffeic acid	
15	4.671	637.1064	637.1046	−2.76	193 (10), 285 (61), 351 (87), 637 (100)	C_27_H_26_O_18_	Flavone	Luteolin-7-diglucuronide	[2,7,8,21,22]
16	4.758	639.1941	639.1931	−1.62	161 (22), 179 (15), 459 (8), 487 (9), 529 (5), 621 (46), 639 (100)	C_29_H_36_O_16_	Hydroxycinnamic acid conjugated to phenylethanoid	Hydroxy(iso)verbascoside (I)	[7,8,21]
17	4.775	163.0399	163.0401	1.03	119 (100)	C_9_H_8_O_3_	Free hydroxycinnamic acid	*p*-coumaric acid	[22]
18	5.078	621.1106	621.1097	−1.40	113 (11), 193 (18), 269 (12), 351 (100)	C_27_H_26_O_17_	Flavone	Apigenin-7-diglucuronide	[7,8,22]
19	5.132	639.1934	639.1931	−0.53	161 (11), 179 (10), 251 (9), 323 (13), 459 (10), 487 (8), 529 (6), 621 (47), 639 (100)	C_29_H_36_O_16_	Hydroxycinnamic acid conjugated to phenylethanoid	Hydroxy(iso)verbascoside (II)	[7,8,21]
20	5.462	651.1226	651.1203	−3.54	193 (9), 351 (100)	C_28_H_28_O_18_	Flavone	Chrysoeriol-7-diglucuronide	[2,8,21,22]
21	5.615	623.2001	623.1981	−3.13	161 (33), 315 (1), 461 (8), 623 (100)	C_29_H_36_O_15_	Hydroxycinnamic acid conjugated to phenylethanoid	Verbascoside/Isoverbascoside/Forsythoside A (I)	[2,7,8,21,22]
22	5.838	491.0839	491.0831	−1.59	300 (35), 315 (100)	C_22_H_20_O_13_	Flavonol	Isorhamnetin-7-*O*-glucuronide	[22]
23	5.965	623.2000	623.1981	−2.97	161 (20), 315 (1), 461 (8), 623 (100)	C_29_H_36_O_15_	Hydroxycinnamic acid conjugated to phenylethanoid	Verbascoside/Isoverbascoside/Forsythoside A (II)	[2,7,8,21,22]
24	6.318	637.2146	637.2138	−1.26	175 (30), 193 (8), 315 (3), 461 (27), 637 (100)	C_30_H_38_O_15_	Hydroxycinnamic acid conjugated to phenylethanoid	Eukovoside (I)	[2,7,8,21,22]
25	6.462	475.0886	475.0882	−0.79	113 (39), 175 (7), 284 (37), 299 (100)	C_22_H_20_O_12_	Flavone	Trihydroxymethoxyflavone glucuronide	
26	6.615	635.1258	635.1254	−0.66	113 (8), 175 (9), 193 (13), 285 (7), 351 (100), 461 (5)	C_28_H_28_O_17_	Flavone	Acacetin-7-diglucuronide	[2,8,21]
27	6.768	637.2145	637.2138	−1.10	161 (10), 175 (28), 285 (6), 315 (3), 351 (9), 461 (18), 637 (100)	C_30_H_38_O_15_	Hydroxycinnamic acid conjugated to phenylethanoid	Eukovoside (II)	[2,7,8,21,22]
28	7.212	651.2306	651.2294	−1.77	175 (73), 193 (9), 475 (5), 591 (18), 651 (100)	C_31_H_40_O_15_	Hydroxycinnamic acid conjugated to phenylethanoid	Martynoside (I)	[2,7,8,21,22]
29	7.592	651.2304	651.2294	−1.46	175 (23), 193 (8), 265 (6), 475 (5), 505 (3), 591 (18), 651 (100)	C_31_H_40_O_15_	Hydroxycinnamic acid conjugated to phenylethanoid	Martynoside (II)	[2,8,21,22]
30	7.772	315.0516	315.0510	−1.81	300 (100), 301 (33)	C_16_H_12_O_7_	Flavone	Eupafolin (6-Methoxyluteolin)	[1]
31	8.312	299.0563	299.0561	−0.62	284 (100)	C_16_H_12_O_6_	Flavone	Trihydroxymethoxyflavone: Hispidulin/Diosmetin/Chrysoeriol	[1]
32	8.415	329.0678	329.0667	−3.41	271 (12), 299 (61), 314 (100)	C_17_H_14_O_7_	Flavone	Trihydroxydimethoxyflavone: Jaceosidin/Cirsilol	[1,2]
33	8.712	313.0715	313.0718	0.84	283 (100), 284 (59), 298 (63)	C_17_H_14_O_6_	Flavone	Dihydroxydimethoxyflavone: Cirsimaritin/Pectolinarigenin	[1]
34	8.802	343.0834	343.0823	−3.12	313 (74), 328 (100)	C_18_H_16_O_7_	Flavone	Eupatorin (3′,5-Dihydroxy-4′,6,7-trimethoxyflavone)	[1]

**Table 4:** Chromatographic peak areas (×10^3^) and heat map obtained by UHPLC-ESI-QTOF-MS analysis of lemon verbena infusions from different commercial brands.

Peak	Compound	Peak Areas			
		A	B	C	D
1	Dihydroxybenzoic acid glucoside	33.7 ± 2.6 ^c^	69.8 ± 8.3 ^b^	115.7 ± 6.9 ^a^	71.5 ± 5.5 ^b^
2	Gardoside (I)	59.5 ± 2.3 ^c^	228.3 ± 11.9 ^b^	320.4 ± 41.7 ^a^	247.6 ± 20.9 ^ab^
3	Gardoside-*O*-methylglucuronide	26.3 ± 4.2 ^b^	149.9 ± 6.1 ^a^	153.5 ± 15.6 ^a^	149.4 ± 13.4 ^a^
4	Shanzhiside	180.1 ± 1.5 ^c^	561.6 ± 31.9 ^b^	639.2 ± 57.7 ^ab^	700.3 ± 32.8 ^a^
5	Ixoside	56.4 ± 3.5 ^c^	233.8 ± 10.5 ^b^	399.6 ± 16.9 ^a^	285.4 ± 39.3 ^b^
6	Gardoside (II)	563.9 ± 38.9 ^c^	1385.9 ± 11.4 ^b^	1539.4 ± 87.7 ^ab^	1587.0 ± 33.1 ^a^
7	Hydroxybenzoic acid-*O*-glucoside (I)	15.4 ± 1.1 ^b^	53.0 ± 2.8 ^a^	56.4 ± 3.0 ^a^	60.9 ± 3.1 ^a^
8	Verbasoside	472.2 ± 41.6 ^b^	1448.9 ± 41.3 ^a^	1602.3 ± 115.4 ^a^	1532.9 ± 101.2 ^a^
9	Hydroxybenzoic acid-*O*-glucoside (II)	44.9 ± 2.5 ^c^	125.0 ± 17.3 ^b^	188.9 ± 12.9 ^ab^	256.8 ± 22.9 ^a^
10	Cistanoside F	644.7 ± 74.4 ^b^	1067.7 ± 22.3 ^a^	1076.3 ± 69.2 ^a^	950.2 ± 62.8 ^a^
11	Caffeic acid-*O*-hexoside (I)	22.6 ± 2.7 ^b^	81.0 ± 11.3 ^a^	104.0 ± 6.9 ^a^	85.2 ± 9.3 ^a^
12	Caffeic acid-*O*-hexoside (II)	34.3 ± 2.1 ^b^	129.1 ± 11.1 ^a^	156.6 ± 4.7 ^a^	133.8 ± 17.4 ^a^
13	Theveside	707.2 ± 91.7 ^b^	951.5 ± 48.3 ^ab^	1177.1 ± 154.9 ^a^	1287.2 ± 94.0 ^a^
14	Caffeic acid	183.9 ± 28.6 ^b^	469.8 ± 71.0 ^a^	445.7 ± 3.5 ^a^	310.7 ± 24.3 ^ab^
15	Luteolin-7-diglucuronide	3857.9 ± 41.7 ^b^	4365.1 ± 172.0 ^a^	4466.2 ± 135.5 ^a^	4312.5 ± 75.2 ^a^
16	Hydroxy(iso)verbascoside (I)	476.1 ± 18.2 ^b^	971.0 ± 67.5 ^a^	894.5 ± 88.9 ^a^	813.0 ± 43.9 ^a^
17	*p*-coumaric acid	390.3 ± 10.1 ^c^	662.5 ± 67.1 ^b^	nd	735.6 ± 37.9 ^a^
18	Apigenin-7-diglucuronide	879.9 ± 88.1 ^b^	984.2 ± 48.2 ^ab^	1071.2 ± 42.4 ^a^	981.9 ± 15.6 ^ab^
19	Hydroxy(iso)verbascoside (II)	190.5 ± 10.3 ^c^	383.3 ± 26.8 ^a^	334.1 ± 18.4 ^ab^	270.8 ± 2.0 ^b^
20	Chrysoeriol-7-diglucuronide	1750.7 ± 34.4 ^b^	1992.2 ± 48.3 ^a^	1959.4 ± 51.7 ^a^	1917.0 ± 44.8 ^a^
21	Verbascoside/Isoverbascoside/Forsythoside A (I)	5188.3 ± 438.2 ^b^	7347.7 ± 34.3 ^a^	7730.9 ± 116.8 ^a^	7594.3 ± 252.4 ^a^
22	Isorhamnetin-7-*O*-glucuronide	171.6 ± 25.0 ^a^	149.4 ± 8.3 ^a^	158.2 ± 4.5 ^a^	172.5 ± 11.9 ^a^
23	Verbascoside/Isoverbascoside/Forsythoside A (II)	4957.7 ± 548.4 ^b^	8938.7 ± 21.4 ^a^	9092.4 ± 332.6 ^a^	8460.5 ± 161.6 ^a^
24	Eukovoside (I)	430.5 ± 27.3 ^b^	984.9 ± 43.4 ^a^	959.4 ± 90.6 ^a^	984.2 ± 103.1 ^a^
25	Trihydroxymethoxyflavone glucuronide	102.0 ± 9.5 ^a^	103.4 ± 5.5 ^a^	105.7 ± 7.2 ^a^	113.9 ± 6.2 ^a^
26	Acacetin-7-diglucuronide	244.2 ± 34.0 ^a^	272.9 ± 25.3 ^a^	267.1 ± 28.5 ^a^	252.9 ± 9.9 ^a^
27	Eukovoside (II)	134.7 ± 3.2 ^b^	227.3 ± 12.1 ^a^	230.2 ± 18.7 ^a^	186.5 ± 24.2 ^ab^
28	Martynoside (I)	448.3 ± 44.5 ^b^	827.5 ± 53.1 ^a^	952.9 ± 34.6 ^a^	812.7 ± 107.1 ^a^
29	Martynoside (II)	90.1 ± 14.4 ^b^	176.6 ± 12.1 ^a^	179.2 ± 0.2 ^a^	117.4 ± 8.8 ^b^
30	Eupafolin (6-Methoxyluteolin)	40.2 ± 5.0 ^b^	99.2 ± 0.5 ^a^	68.7 ± 5.2 ^ab^	44.5 ± 7.9 ^b^
31	Trihydroxymethoxyflavone: Hispidulin/Diosmetin/Chrysoeriol	68.8 ± 0.5 ^c^	157.2 ± 0.8 ^a^	119.4 ± 11.6 ^b^	92.4 ± 6.4 ^bc^
32	Trihydroxydimethoxyflavone: Jaceosidin/Cirsilol	106.9 ± 2.8 ^b^	241.0 ± 10.2 ^a^	227.8 ± 2.8 ^a^	133.7 ± 17.6 ^b^
33	Dihydroxydimethoxyflavone: Cirsimaritin/Pectolinarigenin	45.3 ± 2.5 ^c^	99.2 ± 13.4 ^ab^	113.6 ± 8.8 ^a^	64.7 ± 9.6 ^bc^
34	Eupatorin (3′.5-Dihydroxy-4′.6.7-trimethoxyflavone)	50.9 ± 3.3 ^c^	128.4 ± 11.1 ^a^	129.7 ± 2.7 ^a^	90.1 ± 9.9 ^b^
	**Total**	**22,669.9**	**36,067.0**	**37,035.8**	**35,809.9**
		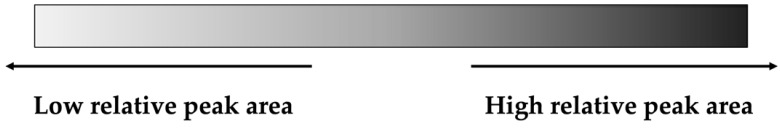			
% Phenolic acids		58.6	62.4	60.8	61.0
% Hydroxybenzoic acids		0.4	0.7	1.0	1.1
% Hydroxycinnamic acids		58.2	61.7	59.8	59.9
% Free Hydroxycinnamic acids		5.6	6.7	4.8	6.2
% Hydroxycinnamic acids conjugated to phenylethanoids		52.6	55.1	55.0	53.7
% Flavonoids		32.3	23.8	23.5	22.8
% Flavonols		0.8	0.4	0.4	0.5
% Flavones		31.5	23.4	23.0	22.4
% Phenylethanoids		2.1	4.0	4.3	4.3
% Iridoids		7.0	9.7	11.4	11.9

For each commercial brand, the chromatographic peak areas are expressed as mean ± standard deviation (1 × 10^3^) of the infusions prepared in triplicate. Within each line, different letters represent significant differences between commercial brands at *p* < 0.05; nd, not detected.

**Table 5:** Free amino acid profile of lemon verbena infusions (ng/mL) from different commercial brands, detected and quantified by RP-HPLC-FLD.

Free Amino Acid	Commercial Brand			
	A	B	C	D
Aspartic Acid	2.98 ± 0.04 ^a^	2.70 ± 0.12 ^b^	2.73 ± 0.05 ^b^	2.72 ± 0.06 ^b^
Glutamic Acid	2.61 ± 0.17 ^a^	2.59 ± 0.08 ^a^	2.52 ± 0.07 ^a^	2.37 ± 0.01 ^a^
Asparagine	3.10 ± 0.48 ^a^	2.38 ± 0.03 ^a^	2.55 ± 0.51 ^a^	2.01 ± 0.22 ^a^
Serine	1.80 ± 0.10 ^a^	1.70 ± 0.04 ^a^	1.75 ± 0.13 ^a^	1.65 ± 0.02 ^a^
Glutamine	1.77 ± 0.14 ^a^	2.01 ± 0.17 ^a^	1.32 ± 0.07 ^b^	1.17 ± 0.11 ^b^
Histidine	nd	nd	nd	nd
Glycine	0.33 ± 0.03 ^a^	0.28 ± 0.03 ^a^	0.29 ± 0.02 ^a^	0.27 ± 0.01 ^a^
Threonine	1.35 ± 0.03 ^a^	1.33 ± 0.01 ^a^	1.35 ± 0.05 ^a^	1.30 ± 0.02 ^a^
Arginine	1.34 ± 0.13 ^a^	1.26 ± 0.06 ^a^	1.43 ± 0.21 ^a^	1.32 ± 0.04 ^a^
Alanine	1.25 ± 0.08 ^a^	1.27 ± 0.03 ^a^	1.32 ± 0.03 ^a^	1.22 ± 0.03 ^a^
Tyrosine	nd	nd	0.06 ± 0.00	nd
Valine	0.93 ± 0.09 ^ab^	0.95 ± 0.05 ^ab^	1.10 ± 0.13 ^a^	0.79 ± 0.05 ^b^
Methionine	nd	nd	nd	nd
Tryptophan	nd	nd	nd	nd
Phenylalanine	0.50 ± 0.04 ^b^	0.71 ± 0.04 ^a^	0.71 ± 0.07 ^a^	0.69 ± 0.03 ^a^
Isoleucine	0.77 ± 0.05 ^a^	0.80 ± 0.03 ^a^	0.83 ± 0.02 ^a^	0.79 ± 0.03 ^a^
Leucine	nd	0.62 ± 0.05 ^a^	0.72 ± 0.06 ^a^	0.64 ± 0.03 ^a^
Lysine	nd	nd	nd	nd
Hydroxyproline	nd	nd	0.13 ± 0.02	nd
Proline	3.06 ± 0.35 ^a^	2.97 ± 0.54 ^a^	2.64 ± 0.62 ^a^	2.05 ± 0.04 ^a^

For each commercial brand, detected concentrations of each amino acid are expressed as mean ± standard deviation of the infusions prepared in triplicate. Within each line, different letters represent significant differences between commercial brands at *p* < 0.05; nd, not detected.

**Table 6:** Pearson correlations were obtained between the detected contents of free amino acids and the results obtained in the spectrophotometric assays.

Free Amino Acid	Spectrophotometric Assay				
	DPPH^•^ Scavenging	FRAP	ORAC	Total Phenolic Content	Total Flavonoid Content
Aspartic Acid	−0.996 **	−0.928	−0.938	−0.959 *	−0.954 *
Glutamic Acid	−0.524	−0.400	−0.129	−0.485	−0.498
Asparagine	−0.873	−0.684	−0.654	−0.771	−0.770
Serine	−0.764	−0.519	−0.543	−0.623	−0.621
Glutamine	−0.328	−0.369	0.067	−0.402	−0.423
Histidine	-	-	-	-	-
Glycine	−0.959 *	−0.822	−0.789	−0.887	−0.886
Threonine	−0.467	−0.167	−0.210	−0.291	−0.291
Arginine	−0.055	0.246	−0.186	0.167	0.186
Alanine	0.263	0.562	0.423	0.452	0.453
Tyrosine	-	-	-	-	-
Valine	0.056	0.369	0.268	0.249	0.249
Methionine	-	-	-	-	-
Tryptophan	-	-	-	-	-
Phenylalanine	0.992 **	0.969 *	0.938	0.986 *	0.982 *
Isoleucine	0.679	0.888	0.683	0.826	0.829
Leucine	0.989 *	0.976 *	0.890	0.996 **	0.995 **
Lysine	-	-	-	-	-
Hydroxyproline	-	-	-	-	-
Proline	−0.557	−0.436	−0.168	−0.520	−0.533

* Correlation is significant at the 0.05 level (two-tailed). ** Correlation is significant at the 0.01 level (two-tailed).

## Data Availability

Not applicable.
